# *SMARCA4* mutations in *KRAS*‐mutant lung adenocarcinoma: a multi‐cohort analysis

**DOI:** 10.1002/1878-0261.12831

**Published:** 2020-12-17

**Authors:** Liang Liu, Tamjeed Ahmed, William J. Petty, Stefan Grant, Jimmy Ruiz, Thomas W. Lycan, Umit Topaloglu, Ping‐Chieh Chou, Lance D. Miller, Gregory A. Hawkins, Martha A. Alexander‐Miller, Stacey S. O’Neill, Bayard L. Powell, Ralph B. D’Agostino, Reginald F. Munden, Boris Pasche, Wei Zhang

**Affiliations:** ^1^ Center for Cancer Genomics and Precision Oncology Wake Forest Baptist Comprehensive Cancer Center Winston‐Salem NC USA; ^2^ Department of Cancer Biology Wake Forest School of Medicine Winston‐Salem NC USA; ^3^ Department of Internal Medicine‐Section of Hematology and Oncology Wake Forest School of Medicine Winston‐Salem NC USA; ^4^ Department of Biochemistry Wake Forest School of Medicine Winston‐Salem NC USA; ^5^ Department of Microbiology and Immunology Wake Forest School of Medicine Winston‐Salem NC USA; ^6^ Department of Pathology and Laboratory Medicine Wake Forest School of Medicine Winston‐Salem NC USA; ^7^ Department of Biostatistical Sciences Wake Forest School of Medicine Winston‐Salem NC USA; ^8^ Department of Radiology Wake Forest School of Medicine Winston‐Salem NC USA

**Keywords:** immunotherapy, KRAS, lung adenocarcinoma, nonimmunotherapy, prognostics biomarker, SMARCA4 mutation

## Abstract

*KRAS* is a key oncogenic driver in lung adenocarcinoma (LUAD). Chromatin‐remodeling gene *SMARCA4* is comutated with *KRAS* in LUAD; however, the impact of *SMARCA4* mutations on clinical outcome has not been adequately established. This study sought to shed light on the clinical significance of *SMARCA4* mutations in LUAD. The association of *SMARCA4* mutations with survival outcomes was interrogated in four independent cohorts totaling 564 patients: *KRAS*‐mutant patients with LUAD who received nonimmunotherapy treatment from (a) The Cancer Genome Atlas (TCGA) and (b) the MSK‐IMPACT Clinical Sequencing (MSK‐CT) cohorts; and *KRAS*‐mutant patients with LUAD who received immune checkpoint inhibitor‐based immunotherapy treatment from (c) the MSK‐IMPACT (MSK‐IO) and (d) the Wake Forest Baptist Comprehensive Cancer Center (WFBCCC) immunotherapy cohorts. Of the patients receiving nonimmunotherapy treatment, in the TCGA cohort (*n* = 155), *KRAS*‐mutant patients harboring *SMARCA4* mutations (KS) showed poorer clinical outcome [*P* = 6e‐04 for disease‐free survival (DFS) and 0.031 for overall survival (OS), respectively], compared to *KRAS*‐*TP53* comutant (KP) and *KRAS*‐only mutant (K) patients; in the MSK‐CT cohort (*n* = 314), KS patients also exhibited shorter OS than KP (*P *= 0.03) or K (*P *= 0.022) patients. Of patients receiving immunotherapy, KS patients consistently exhibited the shortest progression‐free survival (PFS; *P *= 0.0091) in the MSK‐IO (*n* = 77), and the shortest PFS (*P *= 0.0026) and OS (*P* = 0.0014) in the WFBCCC (*n* = 18) cohorts, respectively. Therefore, mutations of *SMARCA4* represent a genetic factor leading to adverse clinical outcome in lung adenocarcinoma treated by either nonimmunotherapy or immunotherapy.

## Graphical abstract

In this work, we study the survival outcomes of patients with lung adenocarcinoma in four independent cohorts. By classifying patients with *KRAS* mutations into three subgroups based on their mutation status of *TP53* and *SMARCA4*, our analysis indicates that patients harboring both *KRAS* and *SMARCA4* mutations do not benefit from the treatment with nonimmunotherapy or immune checkpoint inhibitor‐based immunotherapy. Alternative treatment strategy is requested for this subset of patients.

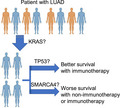

**Abbreviations**
DCB: durable clinical benefit
DFS: disease‐free survival
K: KRAS‐only mutant
KL: KRAS‐STK11 comutant
KP: KRAS‐TP53 comutant
KS: KRAS‐SMARCA4 comutant
LUAD: lung adenocarcinoma
LUSC: lung squamous carcinoma
MSK‐CT: the MSK‐IMPACT clinical sequencing cohort
MSK‐IO: MSK‐IMPACT cohort
NSCLC: non‐small‐cell lung cancer
OS: overall survival
PFS: progression‐free survival
TCGA: The Cancer Genome Atlas
WFBCCC: the Wake Forest Baptist Comprehensive Cancer Center

## 1 Background

Lung cancer is the leading cause of cancer‐related death worldwide, with 5‐year survival rates of ~ 18%. Non‐small‐cell lung cancer (NSCLC) comprises 85% of all lung cancer cases, mainly including adenocarcinoma (LUAD), squamous cell carcinoma (LUSC), and large cell carcinoma. Great strides have been made in recent years with the development of immune checkpoint inhibitor treatment targeting PD‐1/PD‐L1 mediated immunosuppression, which have shown efficacy in up to 30% of NSCLC patients [1, 2, 3, 4, 5, 6]. The expression of PD‐1/PD‐L1 was reported to be associated with enhanced benefits from immunotherapy, but debates exist because of discordant results across different studies [1, 11]. Currently, a higher tumor mutation burden (TMB) is undergoing evaluation as a predictive biomarker in many tumor types [7, 12, 13, 14].

The mutations in *KRAS* are a common oncogenic driver in ~ 20% NSCLC [15, 16]. The goal of developing specific therapeutic strategies for the *KRAS*‐mutant patients has thus far proven elusive. For example, *KRAS* mutations are associated with shortest survivals in NSCLC patients treated with carboplatin plus paclitaxel as well as single anti‐EGFR TKI agent [17]. Recently, it was shown that *STK11*/*LKB1* or *TP53* comutations can stratify *KRAS*‐mutant LUAD patient into different subgroups with distinct biology, therapeutic vulnerabilities and immune profiles [18], and immunotherapy response [19].

The SWItch/Sucrose NonFermentable (SWI/SNF) complex is a major chromatin‐remodeling complex that controls DNA accessibility to transcriptional factors and regulates transcriptional programming [20]. Genomic alterations in the components of the SWI/SNF chromatin‐remodeling complex have been identified in multiple types of cancers [21]. A recent study reported that mutations in the chromatin‐remodeling gene *PBRM1* were associated with response to immunotherapy through IFN‐γ signaling pathway, a key effector for antitumor T‐cell function, in clear cell renal cell carcinoma [22, 23]. Mutations in the *PBRM1* in NSCLC are rare; however, mutations in the *SMARCA4* gene occur frequently in NSCLC [16, 24] and tended to co‐occur with *KRAS* mutations [16]. One recent study showed that *SMARCA4* acted as a tumor suppressor by cooperating with p53 loss and Kras activation, and *SMARCA4*‐mutant tumors were sensitive to inhibition of oxidative phosphorylation [25]. Another study showed that the reduced expression of *SMARCA4* contributes to poor outcomes in lung cancer [26]. However, the prognostic values of *SMARCA4* mutations in *KRAS*‐mutant LUAD patients who received either nonimmunotherapy or immunotherapy treatment have not been well defined.

In this study, we evaluated the prognostic value of *SMARCA4* mutations in *KRAS*‐mutant LUAD within four independent cohorts consisting of patients received nonimmunotherapy or immunotherapy treatment.

## 2 Materials and methods

For the Cancer Genome Atlas (TCGA) cohort, matched somatic mutation, gene expression, and clinical data of 560 patients with LUAD were retrieved. We obtained the clinical and somatic mutation data of 62 principal tumor types for MSK‐IMPACT Clinical Sequencing Cohort and extracted the data of LUAD patients [27]. We excluded patients who received immunotherapy treatment indicated in their later publication [14] (as the MSK‐IO cohort including 186 patients) to establish an MSK‐CT cohort of 1033 patients received nonimmunotherapy treatment.

We extracted the 127 LUAD patients who were treated with immunotherapy between March 1, 2015, and November 30, 2017, at the Wake Forest Baptist Comprehensive Cancer Center (WFBCCC) immune‐oncology program. The experiments were undertaken with the understanding and written consent of each subject, and the study methodologies conformed to the standards set by the Declaration of Helsinki. Efficacy was assessed by the treating physician and categorized according to RECIST guidelines [28] and defined as durable clinical benefit [DCB; complete response (CR)/partial response (PR) or stable disease (SD) that lasted > 6 months] or no durable benefit (NDB, PD, or SD that lasted ≤ 6 months). Progression‐free survival (PFS) was defined as the time from the date of initial immunotherapy administration to the date of progression or death, and overall survival (OS) was to the date of death or last follow‐up, respectively. If the patient was alive at the date of last contact, his/her data were censored at that time point. Genomic profiles were available for 39 patients who were enrolled into the Wake Forest Precision Oncology Initiative (ClinicalTrials.gov Identifier: NCT02566421).

Only patients harboring *KRAS* mutations and with survival data were included in the study, resulting in 155 (27.7% of 560) and 314 (30.4% of 1033) patients received nonimmunotherapy treatment in the TCGA and MSK‐CT cohorts, and 77 (41.4% of 186) and 18 (46.2% of 39) patients received immunotherapy treatment in the MSK‐IO and the WFBCCC cohorts.

### 2.1 Statistical analysis

Tests used to analyze clinical and genomic data included the Mann–Whitney *U*‐test (two‐group comparisons), chi‐square test (three‐group comparisons), and Fisher's exact test (proportion comparisons). Survival curves were estimated using Kaplan–Meier methodology and compared between two groups using the log‐rank test and Cox proportional hazards regression analysis. Hazard ratios (HRs) and 95% CIs were generated by Cox proportional hazards models where *P *< 0.05 and these statistics were estimable (i.e., when at least one event occurred in both groups being compared). All analyses were performed using r software, version 3.2.1 (https://www.r-project.org).

## 3 Results

### 3.1 *SMARCA4* mutations are associated with shorter survival of patients who received nonimmunotherapy treatment

**Fig. 1:**
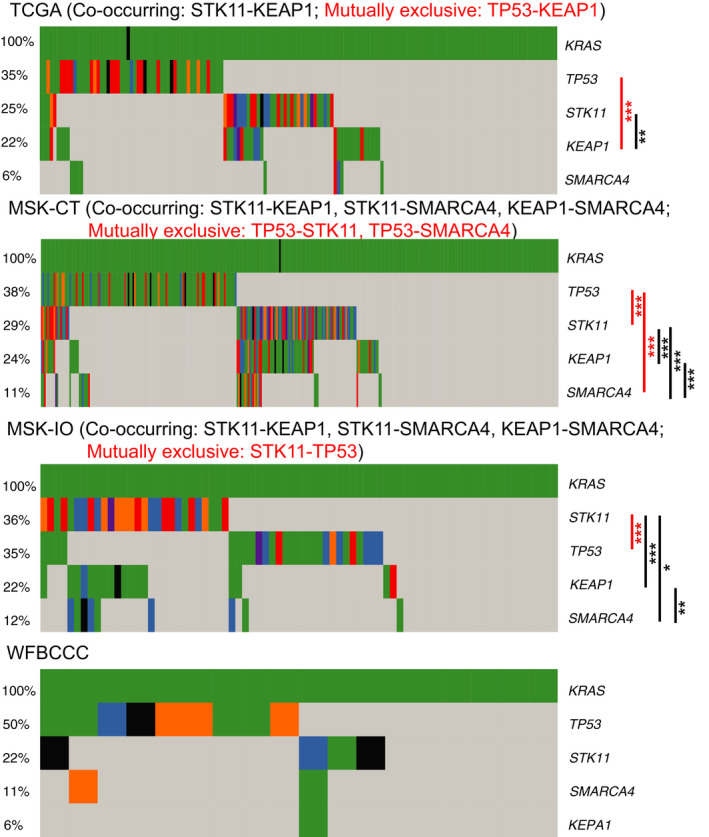
Global somatic mutation landscape of *KRAS*, *TP53, STK11, KEAP1, and SMARCA4* genes in the TCGA, MSK‐CT, MSK‐IO, and WFBCCC cohorts. Comutations were determined with Fisher’s exact test. Black *,**,**: *P *< 0.05, 0.01, 0.001 for co‐occurrence; red ***: *P *< 0.001 for exclusive occurrence.

*KRAS* is one of the most frequently mutated genes in LUAD, which occur in 155 (30%) patients in the TCGA cohort. These patients were reprehensive of the overall LUAD cohort with median patient age of 67 years (range 33–87) and high percentage of current/former smokers (94.8%). 5.8% (9) of the *KRAS*‐mutant patients harbored *SMARCA4* mutations in the TCGA cohort and were classified as KRAS‐SMARCA4 comutant (KS); 33.5% (52) patients harbored *TP53* mutations and were classified as the KRAS‐TP53 comutant (KP) subgroup; and 60.6% (94) patients did not carry *SMARCA4* or *TP53* mutations and were classified as K (Fig. 1). The *SMARCA4* mutations were not associated with any risk factors such as age at diagnosis, tumor stage, race/ethnicity, or smoking history (Table S1).

**Fig. 2:**
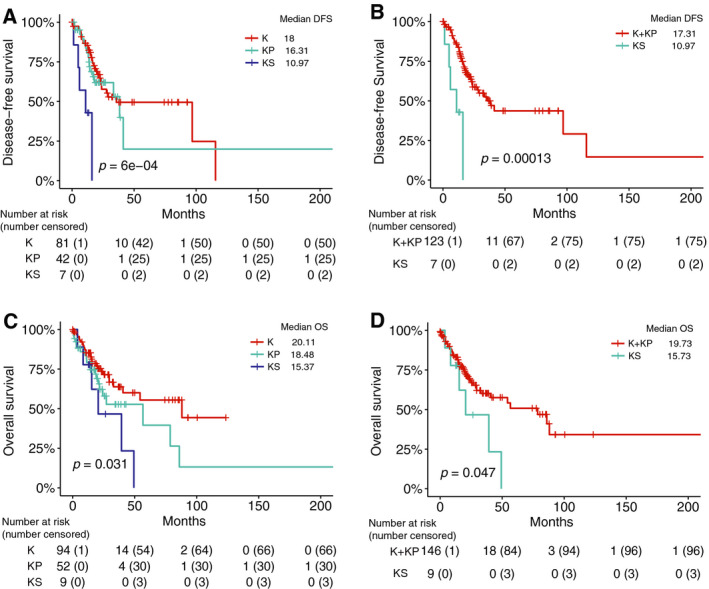
*SMARCA4* mutations are associated with shorter DFS and OS of *KRAS*‐mutant LUAD patients treated with nonimmunotherapy treatment from the TCGA cohort. Kaplan–Meier survival analysis of survival in (A, C) the KS, KP, and K subgroups and (B, D) in the two‐group comparison between *SMRACA4*‐mutant and wild‐type *KRAS*‐mutant patients.

Disease‐free survival (DFS) differed between the three groups (*P* = 6e‐4), with significantly shorter DFS for patients in the KS subgroup compared with either KP (HR 4.47, 95% CI 1.52–13.22, *P *= 0.003) or K (HR 2.43 95% CI 1.46–4.05, *P* = 1.2e‐4) patients in pair‐wise comparisons (Fig. 2A). In contrast, KP and K patient had similar DFS (*P *= 0.64). We also compared the survivals between KS (*SMARCA4*‐mutant) and KP + K (*SMARCA4*‐wild‐type) patients, and found that KS patients exhibited significantly shorter DFS (HR 5.34 95% CI 2.05–14.14, *P* = 1.3e‐4) (Fig. 2B).

Overall survival also varied significantly between the three groups (*P *= 0.031). The KS patients exhibited shorter DFS than the K subgroup (HR 1.63, 95% CI 1.05–2.55, *P *= 0.024). Although the difference in OS between KS and KP was not significant (*P *= 0.21), the median OS in KS was 15.37 months compared with 18.48 months in KP (Fig. 2C). In addition, the two‐group comparison showed significantly shorter OS in KS (*SMARCA4*‐mutant) compared with K + KP (*SMARCA4*‐wild‐type) patients (HR 2.32, 95% CI 1.01–5.44, *P *= 0.047) (Fig. 2D).

**Fig. 3:**
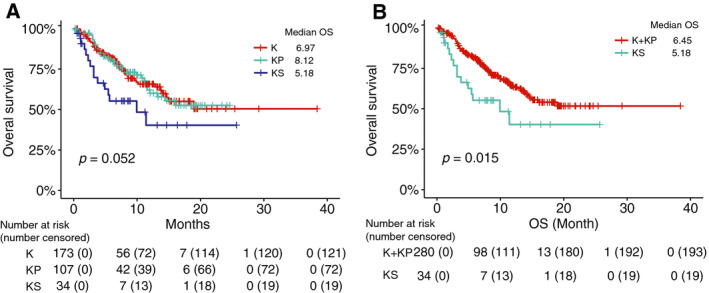
*SMARCA4* mutations are associated with shorter OS of *KRAS*‐mutant LUAD patients treated with nonimmunotherapy treatment from the MSK‐CT cohort. Kaplan–Meier survival analysis of OS (A) in the KS, KP, and K subgroups and (B) in the two‐group comparison between *SMRACA4*‐mutant and wild‐type *KRAS*‐mutant patients.

We validated these observations in an independent MSK‐CT cohort [27], consisting of 314 *KRAS*‐mutant patients. High percentage of current/former smokers (78.0%) were also observed. Across the entire cohort, 10.8% (34) patients were classified as KS, 34.1% (107) were KP, and 55.1% (173) were K (Fig. 1 and Table S2). Significantly shorter OS was observed for patients with KS compared with K (HR 1.39, 95% CI 1.04–1.85, *P *= 0.022) or KP (HR 1.94, 95% CI 1.06–3.57, *P *= 0.03) (Fig. 3A), and K and KP have similar OS (*P *= 0.99). In the two‐group comparison, OS was significantly shorter in KS (*SMARCA4*‐mutant) compared with K + KP (*SMARCA4*‐wild‐type) patients (HR 1.95, 95% CI 1.13–3.38, *P *= 0.015; Fig. 3B).

On the other hand, genes such as *STK11* have been identified as biomarkers for a subgroup (KL) of *KRAS*‐mutant LUAD patients and associated with poorer immunotherapy response [19]. In the TCGA cohort, *SMARCA4* does not show co‐occurring mutations with *STK11*, but the two genes are significantly comutated in the MSK‐CT cohort (Fig. 1). Overall, KS patients experienced the shortest survival in the TCGA (*P *= 0.00028 for DFS and 0.029 for OS; Fig. S1A,B) and MSK‐CT (*P *= 0.038 for OS; Fig. S1C) cohorts, although the differences between KL and KS groups are not significant (*P *> 0.05).

### 3.2 *SMARCA4* mutations are associated with shorter survival of patients who received immunotherapy treatment

We then examined whether *SMARCA4* mutations impacted *KRAS*‐mutant patient response to immunotherapy. Seventy‐seven LUAD patients harboring *KRAS* mutations were extracted from the MSK‐IO cohort [14]. The median age of patients was 68 (range 37–86), and the majority (93.5%) was ever smokers. Based on *SMARCA4* and *TP53* mutation status, 11.7% (9) tumors were classified as KS, 32.5% (25) were KP, and 55.8% (43) were K. Demographic and clinical characteristics were generally well balanced between the comutation defined groups. The clinical benefit rates to immunotherapy in KS, KP, and K groups were not significantly different (*P *= 0.42), probably due to the small sample size; however, smaller proportion of KS patients (2/9 = 22.2%) achieved DCB than KP (10/23 = 43.5%) or K (13/43 = 30.2%) patients (Fig. S1 and Table S3).

**Fig. 4:**
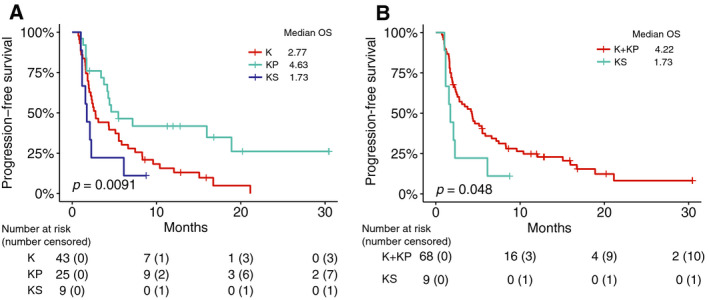
*SMARCA4* mutations are associated with shorter PFS of *KRAS*‐mutant LUAD patients treated with immunotherapy treatment from the MSK‐IO cohort. Kaplan–Meier survival analysis of PFS (A) in the KS, KP, and K subgroups and (B) in the two‐group comparison between *SMRACA4*‐mutant and wild‐type *KRAS*‐mutant patients.

Significantly different PFS was observed between the three groups (*P *= 0.0091). The KS patients exhibited the shorter PFS compared with KP (HR 2.82, 95% CI 1.17–6.81, *P *= 0.016) tumors in pair‐wise comparisons. Although the difference in PFS between KS and K was not significant (*P *= 0.18), the median OS in KS was 1.73 months compared with 2.77 months in KP. Interestingly, KP patients exhibited longer survival than K patients (HR 0.48, 95% CI 0.26–0.86, *P *= 0.012) (Fig. 4A). We merged the KP and K patients to test the difference between *SMARCA4*‐mutant and wild‐type patients. *SMARCA4*‐mutant (KS) patients exhibit significantly shorter PFS than wild‐type (K + KP) patients (HR 2.15, 95% CI 1.46–4.35, *P *= 0.048, median PFS 1.73 vs. 4.22 months) (Fig. 4B).

We also validated the prognostic values of *SMARCA4* mutations in *KRAS*‐mutant LUAD patients upon immunotherapy using 18 patient samples from the WFBCCC. Patients were classified into KS (11.1%), KP (44.4%), and K (44.4%) subgroups (Fig. 1 and Table S4). In this small cohort, the clinical benefit rates to checkpoint inhibitor‐based immunotherapy in KS, KP, and K groups were significantly different (*P *= 0.03). KS patients were resistant to treatment, while KP patients were mostly sensitive.

**Fig. 5:**
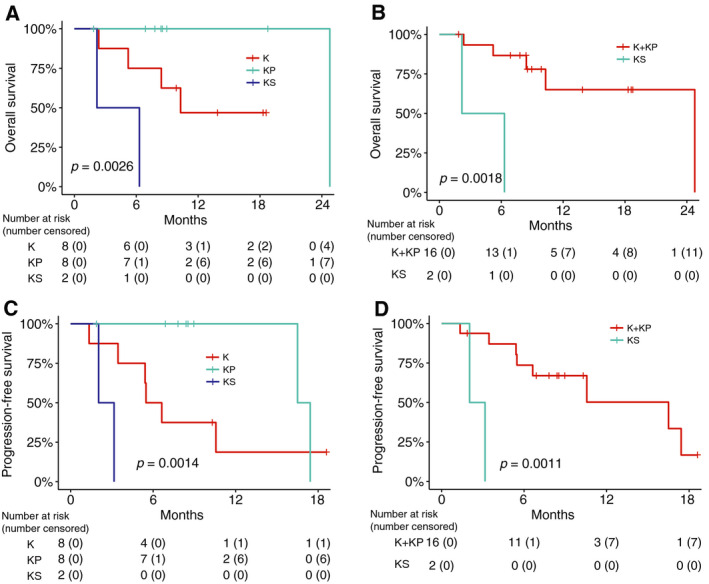
*SMARCA4* mutations are associated with shorter PFS and OS of *KRAS*‐mutant LUAD patients treated with immunotherapy treatment from the WFBCCC cohort. Kaplan–Meier survival analysis of survival (A, C) in the KS, KP, and K subgroups and (B, D) in the two‐group comparison between *SMRACA4*‐mutant and wild‐type *KRAS*‐mutant patients.

The three groups of *KRAS*‐mutant LUAD patients exhibited significantly different OS (*P *=0.042) and PFS (*P *= 0.0014). The KS patients exhibited the shortest OS and PFS compared with either KP (HR 2.46, 95% CI 1.05–6.61, *P *= 0.0019 and *P *= 0.0019 with HR and 95% CI evaluable) and K (HR 2.46, 95% CI 1.01–6.61, *P *= 0.042 and HR 3.06, 95% CI 1.03–10.28, *P *= 0.029) patients in pair‐wise comparisons (Fig. 5A,C). Further significantly deceased OS and PFS were observed in KS (*SMARCA4*‐mutant) patients compared with K + KP (wild‐type) ones (HR 11.98, 95% CI 1.66–26.6, *P *= 0.0018 and HR 18.7, 95% CI 1.65–21.6, *P *= 0.0011) (Fig. 5B,D), consistent with the observations in the MSK‐IO cohort. Altogether, these data indicated that *SMARCA4* abrogation likely determines immunotherapy resistance in *KRAS*‐mutant LUAD.

In addition, analysis showed that *STK11* is comutated with *SMARCA4* in the MSK‐IO cohort but not in the WFBCCC cohort. We further included the KL patient group and tested the survival outcomes of *KRAS*‐mutant patients upon immunotherapy treatment. Consistent with our observation in the analysis of patients who received nonimmunotherapy treatment, KL patients experienced the worst survivals in the MSK‐IO (*P *= 0.036 for PFS; Fig. S1D) and WFBCCC (*P *= 0.00055 for PFS and 7e‐04 for OS; Fig. S1E,F) cohorts.

### 3.3 *SMARCA4* mutations are significantly enriched among tumors with immunosuppressive tumor microenvironment

**Fig. 6:**
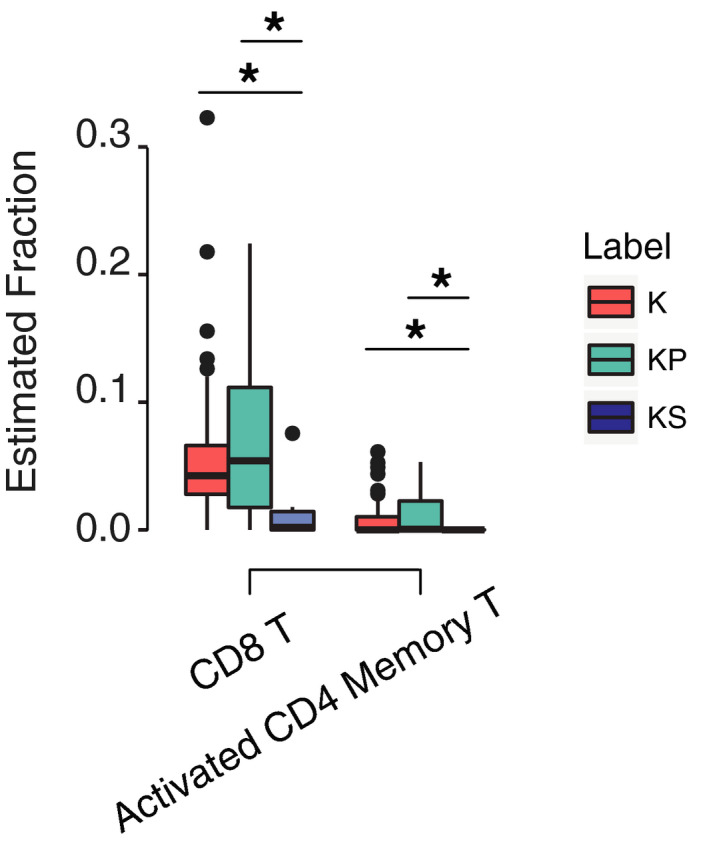
Tumor microenvironment varied among three groups of patients. KS patients contained the lowest proportions of CD8 and activated CD4 memory T cells than either K or KP patients. The plot for all 22 types of immune cells was shown in Fig. S2. *P<.05; Mann–Whitney *U*‐test.

We interrogated the composition of immune cells in the tumor microenvironment of patients from the TCGA cohort which has RNA‐seq data available. Using CIBERSORT [29] and the LM22 signature gene (Table S5) to quantify the proportion of each individual immune cell type, we found that KS patients had significantly lower estimated proportions of CD8 and activated CD4 memory T cells than either K (*P *= 0.015 and 0.035) or KP (*P *= 0.043 and 0.023), indicating an immunosuppressive tumor microenvironment in the KS patients (Figs 6 and S2). We did not observe differences between KP and K patients (*P *= 0.66 and 0.35), which may explain the similar outcomes of these two groups of patients in the TCGA cohort.

## 4 Discussion

Alterations in chromatin‐remodeling complex, SWI/SNF, including *SMARCA4*, have been found in NSCLC [16, 24, 30, 31]. In this study, we interrogated the clinical significance of *SMARCA4* mutations in *KRAS*‐mutant LUAD in the TCGA and the MSK‐CT cohorts in the absence of immunotherapy and the MSK‐IO and the WFBCCC cohorts who received immunotherapy. Our analysis indicates that genomic alterations in the chromatin‐remodeling gene, *SMARCA4*, as a negative prognostic factor to *KRAS*‐mutant LUAD patients no matter received nonimmunotherapy or immunotherapy treatment. The mutations may induce an immunosuppressive tumor environment by modulating the immune cell components. Although the completed determinants of response to treatment are not yet completed defined, our study suggests that nonimmunotherapy and immune checkpoint inhibitor‐based immunotherapy treatment may not benefit this subset of patients.

More frequent *KRAS* mutations were observed in ever smokers than that occurred in never smokers [24, 32, 33, 34], and associated with a significant increase in TMB [35]. Previous studies indicated that a subset of *KRAS*‐mutant NSCLC patients who carry other mutations may have a better response to immunotherapy treatment [2, 35, 36, 37]. We determined that KP patients exhibited better survival than KS and K patients when receiving immune checkpoint inhibitor‐based immunotherapy, which is consistent with previous report [35]. The underlying mechanism may be that KP patients contained the largest proportion of CD8 and activated CD4 memory T cells, supporting by previous report that *TP53* and *KRAS* mutations had remarkable effects on increasing *PD‐L1* expression, facilitating T‐cell infiltration, and augmenting tumor immunogenicity [35]. On the other hand, no improvement of survival was observed in the KS group of patients who received immunotherapy. A possible explanation for this is that the two groups of patients have the similar TMB (Fig. S3), which was shown as a predictive biomarker in many tumor types [7, 12, 13, 14]. Moreover, similar PD‐L1 expression levels were observed between the KL and KS groups of patients (Fig. S4), which suggests their similar outcomes to immune checkpoint inhibitor‐based immunotherapy.

*SMARCA4* inactivation was shown to promote NSCLC aggressiveness by altering chromatin organization [30], and the reduced expression of *SMARCA4* contributes to poor outcomes in lung cancer [26, 38, 39]. *SMARCA4* mutations were distributed throughout the gene and involved most domains (Fig. S5). Here, we showed that *SMARCA4*‐*KRAS* comutant patients (KS) exhibited poorer survival of patients who received either nonimmunotherapy or immunotherapy treatment. On the other hand, quantitative IHC for BRG1 can capture *SMARCA4*‐deficient tumor [40, 41] which is associated with *SMARCA4* mutations (Fig. S6A). Therefore, evaluation of BRG1 expression by IHC may further enhance the predictive utility for nonimmunotherapy or immunotherapy treatment to NSCLC. Further, recent study reported that mutation types determined the expression levels of *SMARCA4*, which is also observed in the TCGA cohort that truncating (nonsense) but not missense mutations, are correlated with the loss of *SMARCA4* expression (Fig. S6B); however, due to the small number of patients carrying nonsense mutations (Fig. S5), the comparison of survivals did not show significant difference (*P *> 0.05; Fig. S6C).

*SMARCA4* mutation is a unique biomarker for the stratification of *KRAS*‐mutant patients with LUAD. Many biomarkers have been reported to stratify patients with LUAD and predict patient outcomes. For instance, *STK11/LKB1* mutations can stratify *KRAS*‐mutant LUAD into different subgroups with distinct biology, therapeutic vulnerabilities and immune profiles [18] and immunotherapy response [19]; however, *STK11* mutations do not serve as a prognostic marker for patients who received nonimmunotherapy treatment [19, 37, 42, 43]. In contrast, *SMARCA4* mutations are associated with shorter survivals of patients who received nonimmunotherapy treatment in both TCGA (*P *= 0.022 for PFS and 0.027 for OS) and MSK‐CT (*P *= 0.0026 for OS) or immunotherapy treatment in the WFBCCC cohort (*P *= 0.012 for OS and 0.0045 for PFS) but not MSK‐IO cohort (*P *= 0.53 for PFS) (Fig. S7). These findings are consistent with a recent publication by Shoenfeld *et al* in terms of *SMARCA4* mutations as poorer prognosis biomarker for patients receiving nonimmunotherapy as well as the comutated genes [44]. Regarding immunotherapy treatment, Shoenfeld *et al*. reported that *SMARCA4*‐mutant patients had better response rates; however, these patients experienced a trend of shorter survivals that is consistent with our observation, although the difference in their analysis is not significant. Also, the four cohorts in our study only consist of patients with LUAD, while the study by Shoenfeld *et al*. covers all subtypes of NSCLC including LUAD, LUSC, and others. Future studies with larger cohort of patients with same histology may be needed.

For these patients harboring both *KRAS* and *SMARCA4* mutations, an alternative treatment strategy is required. A clinical study showed that cisplatin‐based chemotherapy benefited NSCLC patients with low *SMARCA4* expression [26]. Another report indicated the activity of *AURKA*, which encodes a cell‐cycle regulated kinase, was essential in NSCLC cells lacking *SMARCA4*, and the inhibition/depletion of *AURKA* enabled apoptosis and cell death *in vitro* and in xenograft mouse models [45]. As well, CDK4 inhibitor such as Palbociclib may be another option for patients carrying *SMARCA4* mutations [46]. Moreover, a recent study indicated that *SMARCA4*‐deficient lung cells and xenograft tumors displayed marked sensitivity to inhibition of oxidative phosphorylation [25]. All observations suggested encouraging treatment strategies but need further testing in clinics.

## 5 Conclusions

We provide evidence that *SMARCA4* mutations are associated with poor clinical survival outcomes of *KRAS*‐mutant LUAD patients. If confirmed in additional cohorts, it is likely that future prediction models will need to include *SMARCA4* mutations.

## Conflict of interest

The authors declare no conflict of interest.

## Author contributions

LL and WZ designed, analyzed, and interpreted the data. TA, WP, SG, JR, and TL evaluated the clinical data. UT acquired the genomics data. LL and WZ drafted the manuscript. All authors have participated in reading, editing, and approving the final manuscript.

## Consent for publication

Not applicable.

## Ethics approval and consent to participate

Approval for the FoundationOne test was obtained from the ethics committee.

### Peer Review

The peer review history for this article is available at https://publons.com/publon/10.1002/1878‐0261.12831.

## Supporting information


**Table S1.** *KRAS*‐mutant patient Characteristics in the TCGA cohort treated with non‐immunotherapy.
**Table S2.** *KRAS*‐mutant patient Characteristics in the MSKCC‐CT cohort treated with non‐immunotherapy.
**Table S3.** *KRAS*‐mutant patient Characteristics in the MSK‐IO cohort treated with immunotherapy.
**Table S4.** *KRAS*‐mutant patient Characteristics in the WFBCC cohort treated with immunotherapy.
**Fig. S1.** *SMARCA4* mutations are associated with shorter disease‐free survival (DFS) and overall survival (OS) of *KRAS*‐mutant LUAD patients treated with non‐immunotherapy treatment from the (AB) TCGA and (C) MSK‐CT cohorts, and shorter progression‐free survival (PFS) and OS of patients treated with immunotherapy treatment from the (D) MSK‐IO and (EF) WFBCCC cohorts.
**Fig. S2.** The comparisons of estimated proportions of immune cell subsets, as calculated by CIBERSORT among K, KP and KS patients.
**Fig. S3.** The comparisons of TMBs among K, KP and KS patients. ** *P *< 0.01; Mann‐Whitney U test.
**Fig. S4.** The comparisons of PD‐L1 levels among K, KP and KS patients.
**Fig. S5.** Lollipop graph for *SMARCA4* mutations in the four cohorts.
**Fig. S6.** (A) Overall, *SMARCA4* mutations are associated with lower expression level of *SMARCA4*, and (B) Specifically, non‐sense mutations are associated with the lowest expression levels compared to wildtype, missense mutations and other mutations in the TCGA cohort. (C) No significant difference was observed for the patient survivals between those carrying non‐sense and missense/other types of mutations in the MSK‐CT cohort.
**Fig. S7.** *SMARCA4* mutations as a biomarker in LUAD are associated with shorter DFS and OS of patients treated with non‐immunotherapy in the (AB) TCGA and (C) MSK‐CT cohorts, and shorter PFS and OS of patients treated with immunotherapy in the (EF) WFBCCC cohort but (D) not MSK‐IO cohort.

**Table S5.** The LM22 Signature genes, provided at https://cibersort.stanford.edu/download.php.

## Data Availability

All relevant data and materials within this work are made available in this manuscript or previously published.
