# Supplementary material for: SMARCA4 mutations in KRAS‐mutant lung adenocarcinoma: a multi‐cohort analysis
Source: Mol Oncol. 2020 Dec 17;15(2):462–72. doi: 10.1002/1878-0261.12831 (PMC7858279; doi:10.1002/1878-0261.12831)
Supplement: Supplementary file 1 — Table S1. KRAS‐mutant patient Characteristics in the TCGA cohort treated with non‐immunotherapy. Table S2. KRAS‐mutant patient Characteristics in the MSKCC‐CT cohort treated with non‐immunotherapy. Table S3. KRAS‐mutant patient Characteristics in the MSK‐IO cohort treated with immunotherapy. Table S4. KRAS‐mutant patient Characteristics in the WFBCC cohort treated with immunotherapy. Fig. S1. SMARCA4 mutations are associated with shorter disease‐free survival (DFS) and overall survival (OS) of KRAS‐mutant LUAD patients treated with non‐immunotherapy treatment from the (AB) TCGA and (C) MSK‐CT cohorts, and shorter progression‐free survival (PFS) and OS of patients treated with immunotherapy treatment from the (D) MSK‐IO and (EF) WFBCCC cohorts. Fig. S2. The comparisons of estimated proportions of immune cell subsets, as calculated by CIBERSORT among K, KP and KS patients. Fig. S3. The comparisons of TMBs among K, KP and KS patients. ** P < 0.01; Mann‐Whitney U test. Fig. S4. The comparisons of PD‐L1 levels among K, KP and KS patients. Fig. S5. Lollipop graph for SMARCA4 mutations in the four cohorts. Fig. S6. (A) Overall, SMARCA4 mutations are associated with lower expression level of SMARCA4, and (B) Specifically, non‐sense mutations are associated with the lowest expression levels compared to wildtype, missense mutations and other mutations in the TCGA cohort. (C) No significant difference was observed for the patient survivals between those carrying non‐sense and missense/other types of mutations in the MSK‐CT cohort. Fig. S7. SMARCA4 mutations as a biomarker in LUAD are associated with shorter DFS and OS of patients treated with non‐immunotherapy in the (AB) TCGA and (C) MSK‐CT cohorts, and shorter PFS and OS of patients treated with immunotherapy in the (EF) WFBCCC cohort but (D) not MSK‐IO cohort. [file MOL2-15-462-s001.docx]

**Supplementary Figure 1:** *SMARCA4* mutations are associated with shorter disease-free survival (DFS) and overall survival (OS) of *KRAS*-mutant LUAD patients treated with non-immunotherapy treatment from the (AB) TCGA and (C) MSK-CT cohorts, and shorter progression-free survival (PFS) and OS of patients treated with immunotherapy treatment from the (D) MSK-IO and (EF) WFBCCC cohorts. Kaplan-Meier survival analysis was used for the comparison among KS, KP, KL and K subgroups.

** Supplementary Figure 2:** The comparisons of estimated proportions of immune cell subsets, as calculated by CIBERSORT among K, KP and KS patients. Across all cell types, the proportions of CD8 T cells and activated CD4 memory T cells differ significantly in the comparisons of K vs. KS and KP vs. KS (shown in Figure 5).

**Supplementary Figure 3:** The comparisons of TMBs among K, KP and KS patients. ** *P*<.01; Mann-Whitney U test.

**Supplementary Figure 4:** The comparisons of PD-L1 levels among K, KP and KS patients. KL (KRAS-STK11 co-mutation) patients were also included for the comparisons. * *P*<.05; Mann-Whitney U test.


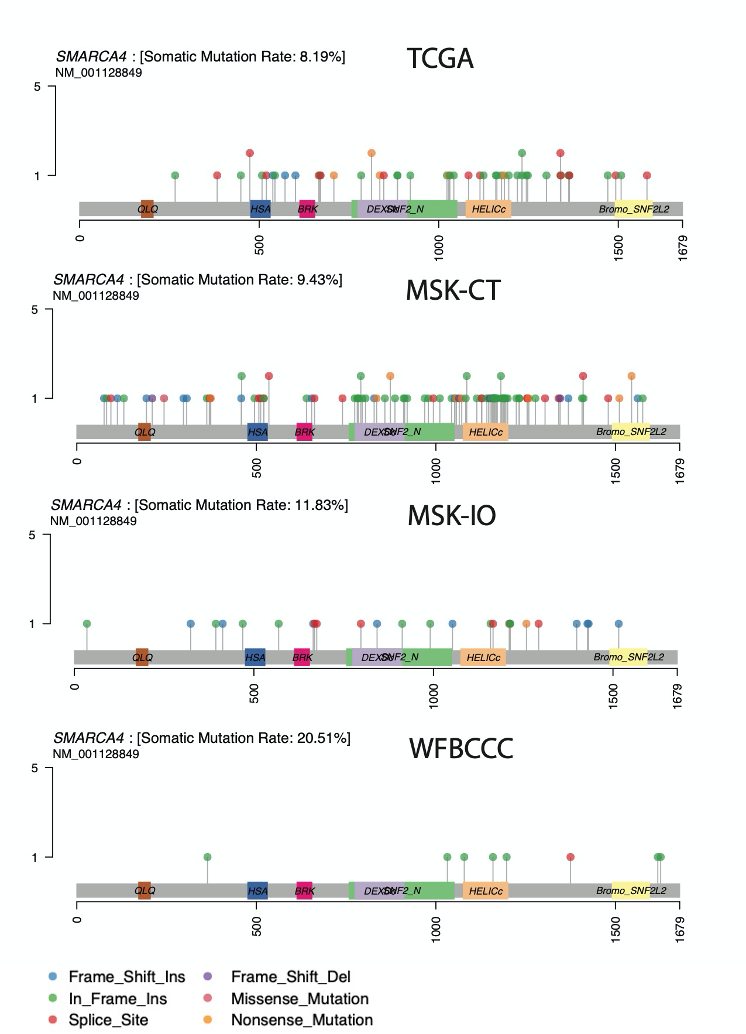


**Supplementary Figure 5:** Lollipop graph for SMARCA4 mutations in the four cohorts.

**
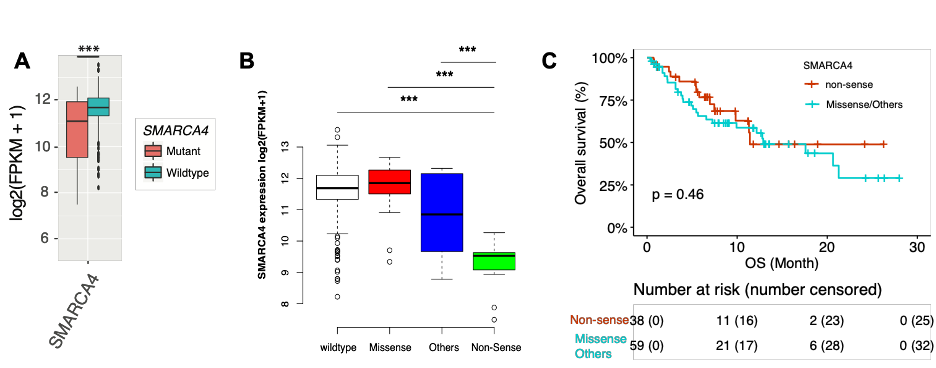
**

**Supplementary Figure 6:** (A) Overall, *SMARCA4* mutations are associated with lower expression level of *SMARCA4*, and (B) Specifically, non-sense mutations are associated with the lowest expression levels compared to wildtype, missense mutations and other mutations in the TCGA cohort. (C) No significant difference was observed for the patient survivals between those carrying non-sense and missense/other types of mutations in the MSK-CT cohort.

**Supplementary Figure 7:** *SMARCA4* mutations as a biomarker in LUAD are associated with shorter DFS and OS of patients treated with non-immunotherapy in the (AB) TCGA and (C) MSK-CT cohorts, and shorter PFS and OS of patients treated with immunotherapy in the (EF) WFBCCC cohort but (D) not MSK-IO cohort.

**Supplementary Table 1**: *KRAS*-mutant patient Characteristics in the TCGA cohort treated with conventional therapy ^a^.

| **Characteristic** | **All** | **K** | **KP** | **KS** | **K vs. KP vs. KS** | **KS vs. K+KP** |
| --- | --- | --- | --- | --- | --- | --- |
| **No. of patients** | 155 | 84 | 52 | 9 |  |  |
| **Median age, years (range)** | 67 (33-87) | 68 (39-87) | 63 (33-83) | 61 (50-71) |  | 0.19 ^b^ |
| **Sex** |  |  |  |  |  |  |
| **Male** | 72 | 46 | 20 | 6 | 0.22 ^c^ | 0.30 ^d^ |
| **Female** | 83 | 48 | 32 | 3 |  |  |
| **Smoking Status** |  |  |  |  |  |  |
| **Ever** | 148 | 89 | 51 | 8 | 0.39 ^c^ | 0.36 ^d^ |
| **Never** | 7 | 5 | 1 | 1 |  |  |
| **Tumor Stage** |  |  |  |  |  |  |
| **I** | 76 | 49 | 24 | 3 |  | 0.62 ^d^ |
| **II** | 40 | 22 | 14 | 4 |  |  |
| **III** | 27 | 15 | 10 | 2 |  |  |
| **IV** | 9 | 5 | 4 | 0 |  |  |
| **Unknown** | 3 | 3 | 0 | 0 |  |  |
| **Race/Ethinity** |  |  |  |  |  |  |
| **White** | 118 | 72 | 37 | 9 |  | 0.39 ^d^ |
| **Black or African American** | 14 | 6 | 8 | 0 |  |  |
| **Asian** | 3 | 2 | 1 | 0 |  |  |
| **Unknown** | 20 | 14 | 6 | 0 |  |  |

| ^a^ Values are reported as No. unless otherwise indicated. Missing values are excluded from the test calculations. |
| --- |
| ^b^ Mann-Whitney U test |
| ^c^ Chi-square test |
| ^d^ Fisher's exact test |

**Supplementary Table 2:** *KRAS*-mutant patient Characteristics in the MSKCC-CT cohort treated with conventional therapy ^a^.

| **Characteristic** | **All** | **K** | **KP** | **KS** | **K vs. KP vs. KS** | **KS vs. K+KP** |
| --- | --- | --- | --- | --- | --- | --- |
| **No. of patients** | 314 | 173 | 107 | 34 |  |  |
| **Sex** |  |  |  |  |  |  |
| **Male** | 121 | 69 | 41 | 11 | 0.71 ^b^ | 0.46 ^c^ |
| **Female** | 193 | 104 | 66 | 23 |  |  |
| **Smoking Status** |  |  |  |  |  |  |
| **Ever** | 245 | 132 | 86 | 27 | 0.89 ^b^ | 1 ^c^ |
| **Never** | 17 | 10 | 5 | 2 |  |  |
| **Unknown** | 52 | 31 | 16 | 5 |  |  |

| ^a^ Values are reported as No. unless otherwise indicated. Missing values are excluded from the test calculations. |
| --- |
| ^b^ Chi-square test |
| ^c^ Fisher's exact test |

**Supplementary Table 3:** *KRAS*-mutant patient Characteristics in the MSK-IO cohort treated with immunotherapy ^a^.

| **Characteristic** | **All** | **K** | **KP** | **KS** | **K vs. KP vs. KS** | **KS vs. K+KP** |
| --- | --- | --- | --- | --- | --- | --- |
| **No. of patients** | 77 | 43 | 25 | 9 |  |  |
| **Median age, years (range)** | 68 (37-86) | 67 (37-86) | 68 (50-83) | 71 (58-81) |  | 0.10 ^b^ |
| **Sex** |  |  |  |  |  |  |
| **Male** | 29 | 14 | 12 | 6 | 0.12 ^c^ | 0.15 ^d^ |
| **Female** | 48 | 29 | 13 | 3 |  |  |
| **Smoking Status** |  |  |  |  |  |  |
| **Ever** | 72 | 39 | 25 | 8 | 0.27 ^c^ | 0.47 ^d^ |
| **Never** | 5 | 4 | 0 | 1 |  |  |
| **Treatment** |  |  |  |  |  |  |
| **PD-(L)1, monotherapy** | 76 | 39 | 19 | 9 | 0.10 ^c^ | 0.60 ^d^ |
| **PD-(L)1 + CTLA-4 combination therapy** | 40 | 4 | 6 | 0 |  |  |
| **Clinical benefit** |  |  |  |  |  |  |
| **DCB** | 25 | 13 | 10 | 2 | 0.42 ^c^ | 0.71 ^d^ |
| **NDB** | 50 | 30 | 13 | 7 |  |  |
| **Not evalable (<6 months follow-up)** | 3 | 0 | 2 | 0 |  |  |

| ^a^ Values are reported as No. unless otherwise indicated. Missing values are excluded from the test calculations. |
| --- |
| ^b^ Mann-Whitney U test |
| ^c^ Chi-square test |
| ^d^ Fisher's exact test |

**Supplementary Table 4:** *KRAS*-mutant patient Characteristics in the WFBCC cohort treated with immunotherapy ^a^.

| **Characteristic** | **All** | **K** | **KP** | **KS** | **K vs. KP vs. KS** | **KS vs. K+KP** |
| --- | --- | --- | --- | --- | --- | --- |
| **No. of patients** | 18 | 8 | 8 | 2 |  |  |
| **Median age, years (range)** | 67 (40-84) | 68 (53-84) | 66 (40-78) | 65 (61-70) |  | S |
| **Sex** |  |  |  |  |  |  |
| **Male** | 6 | 2 | 3 | 1 | 0.75 ^c^ | 1 ^d^ |
| **Female** | 12 | 6 | 5 | 1 |  |  |
| **Smoking Status** |  |  |  |  |  |  |
| **Ever** | 16 | 8 | 8 | 2 |  |  |
| **Never** | 0 | 0 | 0 | 0 |  |  |
| **Treatment** |  |  |  |  |  |  |
| **PD-(L)1, monotherapy** | 15 | 7 | 7 | 1 | 0.41 ^c^ | 0.31 ^d^ |
| **PD-(L)1 + CTLA-4 combination therapy** | 3 | 1 | 1 | 1 |  |  |
| **Clinical benefit** |  |  |  |  |  |  |
| **DCB** | 10 | 3 | 7 | 0 | 0.03 ^c^ | 0.18 ^d^ |
| **NDB** | 8 | 5 | 1 | 2 |  |  |

| ^a^ Values are reported as No. unless otherwise indicated. Missing values are excluded from the test calculations. |
| --- |
| ^b^ Mann-Whitney U test |
| ^c^ Chi-square test |
| ^d^ Fisher's exact test |

**Supplementary Table 5:** The LM22 Signature genes, provided at <https://cibersort.stanford.edu/download.php>.
